# Eye gaze in live-electronic music performance: visual information and looking–listening interplay

**DOI:** 10.3389/fpsyg.2026.1855923

**Published:** 2026-09-14

**Authors:** Tim-Tarek Grund, Alex Hofmann

**Affiliations:** Department of Music Acoustics–Wiener Klangstil, mdw–University of Music and Performing Arts Vienna, Vienna, Austria

**Keywords:** auditory perception, eye tracking, live-electronic music performance, scan path analysis, stimulated recall interview, visual information

## Abstract

Gaze tracking in music performance research often pertains to acoustic instrument performance. The differences between live-electronic music performance and acoustic instrument performance warrant an investigation of how performers use gaze in live-electronic music performance. We examine the combined use of gaze tracking recordings of 18 live-electronic performances with post-performance stimulated recall interviews with the goal of gathering qualitative information about gaze and the live-electronics setup. We identify two primary functional categories of gaze: visual information and looking–listening interplay. Visual information entails gathering visual feedback about the state of the setup and locating targets for hand-eye coordination. Beyond visual information gathering, gaze appears to actively shape auditory perception: participants reported that looking at an interface element facilitates retrieval of memory representations of a live-electronics setup, such as mappings or signal paths. Through visuospatial attention, gaze may support auditory information processing within the complex soundscapes of live-electronic music performance.

## 1 Introduction

Imagine you are sitting in the audience at a live-electronic music performance. A performer, hunched over their laptop, is typing and clicking on it, and a soundscape unfolds. With some keystrokes and clicks, nothing changes in the sound. With others, the sound suddenly shifts. The performer may alter parameters of a generative stochastic process that triggers note events in the future. To you, however, they could have been checking their emails. From an outsider perspective, live-electronic music performance can be quite opaque. The designed association from action to sound in electronic devices for music-making, known as mapping, does not need to imitate physical constraints. This facilitates sound effects that are impossible to predict from performer actions. The use of automatic sound playback techniques such as loops or generative processes adds another layer of opacity to this. As an audience's appreciation of a performance can be mediated by an understanding of the material nature and physical constraints of the acoustic instrument (“How does a trumpet sound”), complemented by knowledge of genre (“How does a trumpet sound in jazz”), live-electronics setups (LES) on their own often lack this level of performance transparency. An underlying mapping, although mediated through (platform) idiomaticity, genre, and “craft practices” (McPherson and Tahroğlu, 2020), could at least in theory take any shape or form and might even evolve over the course of a performance. How can we begin to understand a live-electronic music performance if not through knowledge of the idiomatic relationship between action and sound?

One approach to identify relationships between actions, devices for music-making, and their effect on the performance is to investigate the performer's eye gaze in order to identify the momentary focus of visual attention. This can be accomplished through the process of gaze tracking. Gaze tracking is a procedure in which the position of the point of gaze in an environment is estimated from measurements, often accomplished by recording videos of the eyeballs, where the position of the pupil is extracted via image processing. Through this process, the relative position of the point of gaze can be estimated (Shehu et al., 2021). During saccadic eye movements, the eye alternates between moments of gaze on a single location and moments of jumping to a new location. The stationary moments are called fixations, and they are involved in processing information. Therefore, they are usually considered the current point of attention. Saccades are the jumps in between, and they signal a shift in attention (Eckstein et al., 2017; Hayhoe and Ballard, 2005). In the context of this work, we employ a world-centered frame of reference for fixations and saccades, where they are discussed in relation to virtual points in the environment of the performer, not as eye movements (Hessels et al., 2018).

Gaze tracking in acoustic instrument performance often involves investigations of inter-performer communication (Kawase, 2014; Bishop et al., 2019) or sight-reading (Sloboda, 1974). However, directly applying findings from acoustic instrument performance research to live-electronic music performance can be a risky endeavor, as there are fundamental differences in how performers interact with their means of musicking. In electronic music performance, performers interact with a live-electronics setup to generate, trigger, or modify sound. This LES consists of one or more devices. These devices are mapped to sound generators and sound modifiers. Each device contains an interface to the performer. An interface may consist of one or more interface elements, which allow the performer to control the musical system or to receive information on the system's status. Common control interface elements are buttons, knobs, and faders, which are typically operated by hand. Common information interface elements are simple LEDs or visual displays that can present text, images, or even videos. Furthermore, live-electronic music performance may not follow an instrumental interaction paradigm. Instead, they often follow an exploratory performance paradigm, where the performance takes the form of an exploration of the capabilities of a musical system, a particular mapping, or a body of data, rather than an interpretative performance paradigm. Furthermore, as is the case with techniques such as looping, sequencing, and time-stretching, electronic music performance can require a performer to consider events in the now, the past, and the future (Lopes et al., 2017). Jensenius (2022) discusses relationships between temporal levels in a live-electronic music performance produced by these techniques. The effects of musical actions on sound at different temporal levels can affect the mental model of a performance and influence the cognitive load, as they may linger far longer than in acoustic instrument performance. Therefore, we consider an ecologically grounded study an appropriate tool to better understand nuances of different live-electronic music performances.

Furthermore, the field of electronic music is difficult to define. Ciciliani (2014) considers four genres of electronic music for their investigation of electronic music performance: “academic electronic music, electronica, improvised electronic music and electronic music with an affinity to media-art.” Emmerson and Smalley (2001) define live-electronic music as the use of technology during a performance setting to generate, trigger, or modify sounds. For them, it is a genre of electro-acoustic music and is separate from acousmatic music, which exists only in a recorded state. DJs and keyboard synthesizer performers may also fit into Emmerson and Smalley's definition. For this work, we therefore decided to adopt Emmerson and Smalley's definition, albeit with additional considerations.

To thoroughly investigate a live-electronic music performance, one can consider eye gaze. To this end, a scan path can be created, which visualizes all fixations and saccades that can be mapped to a reference image. Investigating scan paths helps answer one of the central questions of this study: What parts of a live-electronics setup are given attention? Another potent source of internal information about a live-electronic music performance is the performer. In a study on acousmatic music interpretation, Boutard and Féron (2021) applied a stimulated recall interview method to the documentation of acousmatic music. They started by recording automation data on a sound mixing console during performances. Then, by confronting the performers during the interview with a re-enactment of the performance, they provided suitable conditions for precise feedback on playing technique. Gaze recordings in turn may provide suitable conditions for precise feedback on gaze and attention. While previous research suggests that combining gaze tracking with stimulated recall methods provides data that could not have been gathered by either method on its own (Guan et al., 2006; Hyrskykari et al., 2008), certain aspects can be unreliable: the retrospective nature of the method can generate unreliability, such as in reports of the emotional state of a participant (Bruun and Ahm, 2015). While automated processes are hard to verbalize (Hyrskykari et al., 2008), it can still reveal unconscious allocations of attention to some degree (Heinsen, 2024).

Electronic music performance is a complex task, and performer short-term memory is limited (Miller, 1956; Baddeley, 1994). Because remembering every parameter state of a performance may exceed the limits of memory, visual attention is used to select and receive visual feedback during performance and thus helps in managing memory resources. Eye gaze is often treated as the center of visual attention ((Eckstein et al., 2017; Hayhoe and Ballard, 2005)). This eye gaze can be used to visually partition a continuous performance into segments with alternating attention, which can be useful confrontational material for a stimulated recall interview to aid with reflection. It can also help us separate more relevant from less relevant interface elements. The research question is: What functions does gaze serve in live-electronic music performance, and how can it deepen our understanding of a performance and the associated live-electronics setup?

## 2 Methods

### 2.1 Experimental procedure

We recorded the eye gaze of participants during a live-electronic music performance and subsequently used these recordings with an overlay of momentary gaze position as confrontational material for a stimulated recall interview. Furthermore, we captured additional data using a questionnaire. For capturing the gaze data, we adhered to the following procedure. First, we asked participants to perform 5 to 6 min of music using their own personal live-electronics setups. We recorded their eye gaze and their field of view (FOV) using a mobile eye tracker. In addition, a video of the performance was captured from a different perspective along with audio from the proximity of the performer. The recording from the mobile eye tracker also yielded a video with sound in which the momentary gaze of the participant is displayed as a red circle. Directly after each performance, we used this video as confrontational material in the stimulated recall interview in order to gain reflective statements from the performers about their performance and the role of gaze within it. The video with the momentary gaze displayed on top was shown on a companion device. Participants were asked to watch the gaze tracking video and comment on reasons why their gaze moved from one location to another. Furthermore, they were allowed to stop the playback and rewind the video. They were also asked questions about their setup, individual devices, and their functions by the interviewer (the first author). We use these interviews in two ways. First, we collect statements by the performers on the role of gaze during their performance and group these together to identify common trends. Second, we analyze interviews of selected performances in depth and compare performer statements with data from the gaze recording.

Participants were partly recruited through an open call during Kilele 2024, a symposium for music, music technology, and culture in Nairobi (Kenya) (Guttridge-Hewitt, 2024). Further participants were recruited in Berlin (Germany) and Vienna (Austria). Participants were informed about the procedure, had the opportunity to ask questions, and gave their written consent. Participants took part voluntarily and were able to abort the experiment anytime without any penalty. As a reward for participating, participants received a heatmap of their eye gaze during the performance. The experimental procedure received prior ethical clearance by the host institution's ethics committee.

### 2.2 Experimental equipment

We used the Neon mobile eye tracker (by Pupil Labs) with two infrared cameras at 200 frames per second (FPS) pointed at the pupils and an RGB field of view (FOV) camera at 30 FPS. The eye tracker is pictured in Figure 1A, alongside a field of view recording with a fixation overlay in Figure 1B. A second perspective of the scene was filmed with an Alpha 7S III camera (by Sony), and sound was captured using the H2n recorder (by Zoom). The eye tracker recording yielded the point of gaze (POG) at 200 Hz, which was presented to the participants during the stimulated recall on the companion device, an Edge 40 Pro (by Motorola).

**Figure 1:**
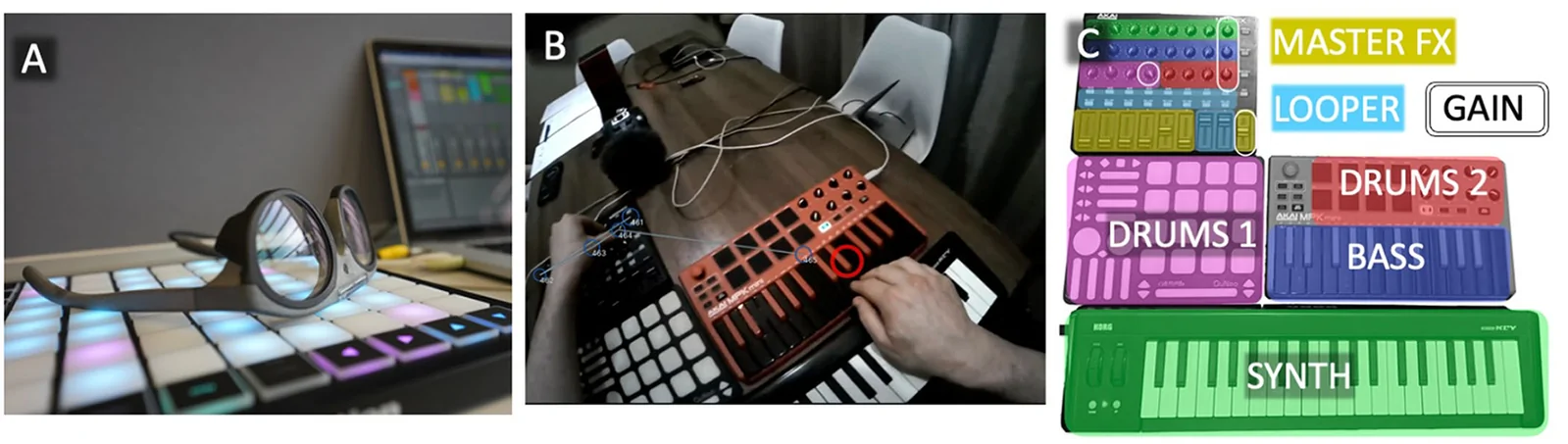
**(A)** The Neon eye tracker (by Pupil Labs). **(B)** The field of view recording from the Neon eye tracker with an overlay of fixations (blue circles, numbered) and saccades (connecting lines between circles). Pictured is a moment, where participant 1 moves from looking at a section of the knob grid on the AKAI Midimix controller (mapped to a bass instrument rack) to the keyboard section on the AKAI MPK Mini, which controls the pitch of the bass instrument rack. **(C)** A mapping visualization of the performance setup of participant 1.

### 2.3 Participants

In this manuscript we will analyze 18 performances by *N* = 18 performers. Four performers reported as female, while 14 reported as male. Participants were on average 32.83 years old (SD = 6.80, Min = 23, Max = 47), with an average of *M* = 8.67 years of self-reported experience with live-electronics (SD = 7.25).

Initially, we had captured 24 performances from 23 participants from different fields of live-electronic music. One participant performed twice with different LES. The latter of these performances is excluded from the dataset. Two participants were excluded from the dataset because they performed in lighting conditions insufficient for mapping gaze to a reference image. Another participant was excluded due to performing from a score. For one participant, the devices were spread out too far to calculate a meaningful scan path; therefore, they were also excluded from the analysis. One more participant did not give consent to appear in a scientific publication. Two participants were seasoned musicians with more than 20 years of experience with acoustic instrument performance but had less than 1 year of experience with LE. However, since one had already performed with an LES and the other had an LE performance planned for the following evening, we decided to include them nonetheless. A list of hardware and software used in the performance paired with a brief description of the performance is shown in Table 1. We gathered supplementary information on participants and their live-electronics setup through a questionnaire.

**Table 1:** List of hardware, software and brief descriptions of the performances of each participant.

ID	Hardware (*n*_*F*_; *t*_*FD*_; μ_*FD*_; ρ_*PD*_)	Software	Brief description
1	AKAI Midimix (401; 03:14 min; 0.484 s; 52.7%), AKAI MPK Mini (127; 00:44 min; 0.347 s; 11.9%), Keith McMillen QuNeo (75; 00:25 min; 0.332 s; 6.8%), Korg Microkey (6; 00:02 min; 0.254 s; 0.5%)	Ableton Live	Midi interfaces control parameters of Ableton Live Plugins. QuNeo controls Drums and XY-Pads, Midimix controls Macro parameters, keyboards control Instrument racks. Music style: experimental improve. Duration *t*_*PD*_: 06:08.334 min.
2	AKAI APC Keys (518; 04:18 min; 0.498 s; 75.8%), Apple laptop (73; 00:46 min; 0.625 s; 13.5%), Zoom U44 (3; 00:02 min;0.532 s; 0.6%)	Ableton Live	Clip Launcher on the APC Keys enables/disables clips. Knobs on APC Keys control parameters. Macbook shows Ableton Live view with enabled clips, volume and other information. Music style: Organic electronic. Duration *t*_*PD*_: 05:40.336 min.
3	Native Instruments TRAKTOR Kontrol S4 (359; 03:33 min; 0.594 s; 43.3%), Apple monitor (356; 03:25 min; 0.576 s; 41.6%)	Native Instruments Traktor Pro 3	DJ Setup with deck with 2 jogwheels and mixer section. Traktor Kontrol S4 controls Traktor Pro (Parallel mode). Music style: House music, Afro house, 3-step. Duration *t*_*PD*_: 08:12.407 min.
4	Laptop (706; 05:46 min; 0.49 s; 87.2%)	Sonic Pi	Live Coding with Sonic Pi. Music style: experimental music. Duration *t*_*PD*_: 06:36.794 min.
5	Apple laptop (315; 03:33 min; 0.678s; 47.1%), Native Intruments KORE 1 (207; 01:46 min; 0.514 s; 23.4%), Ableton Push 1 (139; 00:57 min; 0.407 s; 12.6%)	Ableton Live	Samples are loaded into a granular buffer in Ableton using the Push 1. Kore 1 controls granular parameters, such as playback position. Apple laptop shows waveform, playback position and other performance parameters. Music style: Soundscape, Drone, Ambient, Noise. Duration *t*_*PD*_: 07:32.167 min.
6	M-Audio Keystation 61 Mk 3 (728; 05:40 min; 0.466 s; 72.8%), Apple laptop (56; 00:36 min; 0.643 s; 7.7%)	GarageBand	Keystation keyboard sends MIDI note messages to GarageBand, where they control different keyboard plugins. Music style: R&B, Pop, Electronic. Duration *t*_*PD*_: 07:47.029 min.
9	Ableton Push 3 (standalone) (704; 04:15 min; 0.362 s; 49.5%), Elektron Digitakt (415; 02:31 min; 0.466 s; 29.3%)	Ableton Live (standalone)	Push 3 and Digitakt run in parallel, Push 3 sends MIDI and Clock signal to Digitakt. Music style: melodic/Breakbeat. Duration *t*_*PD*_: 08:34.844 min.
10	Novation Launchcontrol XL (440; 02:43 min; 0.371 s; 50%), Faderfox PC4 (164; 01:05 min; 0.398 s; 19.9%), Monome Grid (16x8) (49; 00:18 min; 0.363 s; 5.5%), Novation Launchpad Mini (35; 00:18 min; 0.524 s; 5.5%), Handheld Clip Microphone (not in view).	Ableton Live, Beatfader, Paulstretch	Launchpad triggers different scenes, and controls recording and playback of vocal sounds through the microphone. Monome Grid sequences percussive guitar sounds. Faders on the Launchcontrol XL control a Beatfader Plugin. Launchcontrol XL also controls other drum plugins, backing vocals, loops and additional processing. PC4 controls parameters in Ableton as well. Music style: experimental electro-acoustic improvisation. Duration *t*_*PD*_: 05:26.242 min.
12	Elektron Digitakt (298; 02:38 min; 0.53 s; 40.9%), Korg Kaoss Pad KP3+ (155; 01:17 min; 0.496 s; 19.9%), Roland SH-01A (117; 01:26 min; 0.731 s; 22.3%)	—	Digitakt sequences SH-01A using MIDI, SH-01A sends Audio to Digitakt. Digitakt then sends Audio to Kaoss Pad, where it is processed. SH-01A keyboard is not used, it is instead controlled with prepared sequences. Music style: Techno. Duration *t*_*PD*_: 06:26.053 min.
13	Arturia Microbrute (568; 04:26 min; 0.469 s; 76.6%), Nord Keyboards Nord Stage 2 EX (38; 00:16 min; 0.431 s; 4.6%)	—	Participant plays bassline on the Microbrute with the left hand, controls Microbrute filter with the right or plays organ chords on the Nord Stage 2. Music style: Jazz/Ska. Duration *t*_*PD*_: 05:47.336 min.
14	Laptop (796; 06:07 min; 0.462 s; 74.9%), AKAI LPK25 (200; 00:44 min; 0.22 s; 9%)	FL studio	Participant produces a beat. First, drum sounds are selected and sequenced in the FL studio step sequencer. Then, piano, strings, synths and bass lines are added. These patterns are then arranged and sound effects are added. Music style: HipHop, Deutschrap. Duration *t*_*PD*_: 08:09.943 min.
15	AKAI MPC3000 (1,156; 07:03 min; 0.366 s; 66.1%), Laptop (180; 01:45 min; 0.583 s; 16.4%)	FL studio	Participant produces a beat. First, drum sounds are selected on the laptop and then recorded into the MPC. Then, a pattern is recorded into the MPC. Drum sounds are tweaked. Subsequently, new percussive and non-percussive sounds are recorded into the MPC, performed, and tweaked. Music style: HipHop/South/Westcoast/90s/2000s. Duration *t*_*PD*_: 10:39.644 min.
17	Arturia Minifreak (693; 05:22 min; 0.464 s; 85.8%)	—	Participant plays chords or long notes with the left hand. The right hand either adds melodic lines with the keys or tweaks the synthesis parameters. Music style: Electronic. Duration *t*_*PD*_: 06:15.289 min.
18	Korg Minilogue xd (467; 02:14 min; 0.289 s; 32.1%), Roland TB-03 (244; 01:13 min; 0.297 s; 17.5%), Apple laptop (131; 00:46 min; 0.351 s; 11%), Arturia APC Keys (64; 00:18 min; 0.286 s; 4.3%), DJ Techtools MIDI Fighter Twister (63; 00:22 min; 0.343 s; 5.3%),	Ableton Live	MIDI fighter twister controls filters and gain of different elements. Left hand plays and tweaks parameters of chord stabs and pads on the Minilogue xd. Clip section from APC Keys controls drum clips and bassline patterns. TB-03 plays and controls basslines. Music style: Jazz, House, Experimental. Duration *t*_*PD*_: 06:57.964 min.
19	DIY Sequencer (356; 03:04 min; 0.517 s; 48.3%), DIY Oscillator (120; 00:38 min; 0.316 s; 10%), DIY Power supply (0; 00:00 min; 0 s; 0%), Behringer XENYX QX1202USB (254; 01:42 min; 0.403 s; 26.8%)	—	DIY sequencer sends electrical signal (not Control Voltage) to the Oscillator to control the pitch. Yellow button on sequencer controls speed, others control pitch of associated step. QX1202 has EQ, compression, gain and FX controls. FX here was a pitch shifter. Oscillator has a triangle waveform and controls for gain, pitch and modulation. Music style: Noise. Duration *t*_*PD*_: 06:20.694 min.
20	Roland MC-707 (691; 03:33 min; 0.308 s; 55%), Roland SP-404 MkII (206; 01:15 min; 0.363 s; 19.4%), Novation Peak (74; 00:23 min; 0.311 s; 5.9%)	—	The MC-707 sends MIDI sequences to the Peak synthesizer, which returns audio to the SP404. The SP-404 is used as a sampler but also FX processor and sends audio back to the MC-707, which sends audio to the speakers. Percussions, basslines, vocals and riseups are done on the MC-707, the SP-404 is used for triggering samples that are organized in sequences. Music style: Psytrance. Duration *t*_*PD*_: 06:27.535 min.
21	Roland SP-404 MkII (1,208; 05:20 min; 0.265 s; 77.5%)	—	The SP-404 is used to play and process pre-recorded samples with pitch, drive and delay effects. Music style: Noise, Ambient. Duration *t*_*PD*_: 06:29.648 min.
22	Apple laptop (547; 04:30 min; 0.493 s; 92.7%)	Pure Data, Cloud Hands, Python	The laptop runs Cloud Hands, a hand landmark detection python script and a granular sample processing patch in Pure Data. The participant selects samples using the laptop's trackpad, and controls volume, playback position and granular parameters using the position and openness of the hands. Music style: Pop, Soul, Blues. Duration *t*_*PD*_: 04:51.405 min.

In total, 46 hardware devices were used by the performers. Fixation Count (*n*_*F*_), Total Fixation Duration (*t*_*FD*_), Average Fixation Duration (μ_*FD*_) and Ratio of Performance Duration (ρ_*PD*_) are presented for each device. Each participant's self-reported music style is reported. The duration of each performance is given as *t*_*PD*_.

### 2.4 Data processing

Video recordings were prepared for data analysis by setting start and end points within Pupil Cloud (by Pupil Labs), a designated software solution for aggregating and visualizing gaze tracking data from the Neon mobile eye tracker (by Pupil Labs). There, fixations were automatically mapped to points on a reference image with manual corrections where necessary. Each area of the reference image that corresponds to a device of an LES was designated as an area of interest for further analysis. Fixation Count *n*_*F*_, Total Fixation Duration *t*_*FD*_, and Average Fixation Duration μ_*FD*_ were extracted by Pupil Cloud from fixations manually assigned to the areas of interest. We calculated the Ratio of Performance Duration ρ_*PD*_ as the ratio of *t*_*FD*_ per area of interest to the performance duration *t*_*PD*_ (see Table 1). μ_*FD*_ is calculated as the Fixation Count *n*_*F*_ divided by the Total Fixation Duration *t*_*FD*_. ρ_*PD*_ is calculated as the Total Fixation Duration *t*_*FD*_of a given area of interest divided by the Performance Duration *t*_*PD*_.

Fixations and saccades that occurred within the performance duration were visualized on the reference image to produce scan path visualizations (see Figure 2). Fixations are displayed as circles, and saccades as the lines that connect two fixation circles. The scan paths produced through the gaze tracking are then analyzed visually. Features of interest of the scan paths are clusters of fixations but also emergent geometric patterns between them that allow for insights to be made about the temporal relationship between devices. Insights about the functional relationship can be drawn by combining the results of the scan path analysis with the results of the thematic analysis.

**Figure 2:**
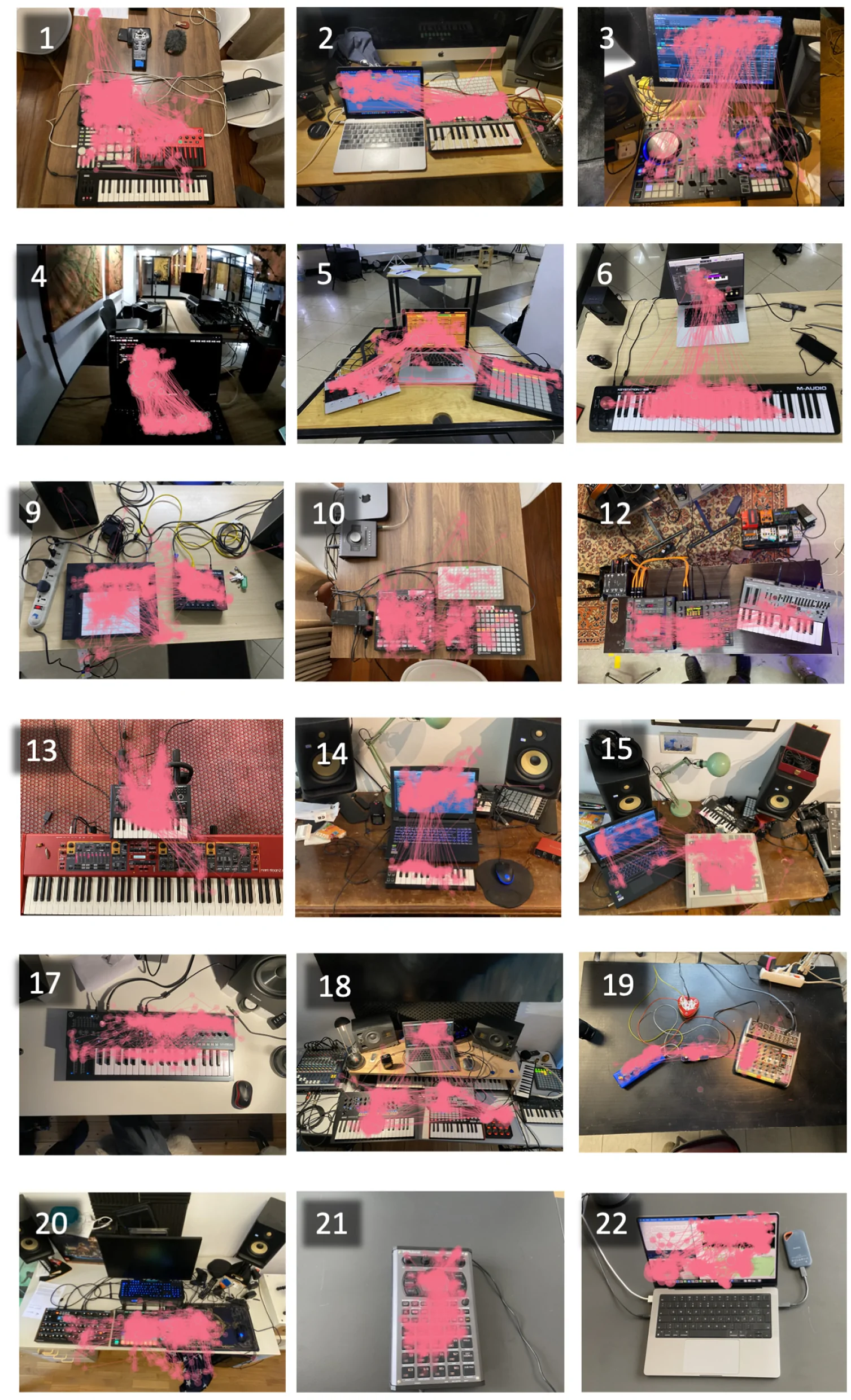
Scanpaths of the performances.

Post-performance stimulated recall interviews were transcribed with the WhisperScript application (by Wavery) and manually corrected. Pupil diameter data are also available; However, due to the limited control over equal lighting conditions across performances, it was decided to discard it. In order to identify recurrent themes among participants, a thematic analysis is carried out, following the guidelines recommended in Ahmed et al. (2025). First, interview transcripts are read multiple times to familiarize ourselves with the data and develop broad topics of interest: looking, hearing, focus, complexity of the system, visual information, and visual feedback. Then, participant statements are marked when they pertain to these topics, and codes are identified. Then, the identified codes are refined into broader themes, but also to develop new potential themes to investigate. The transcripts are then read again to identify more statements that fit these themes. Themes may be combined if there is sufficient thematic overlap.

## 3 Results

### 3.1 Overall findings

Participants that responded to the study call exhibited a wide range of technological means and stylistic expressions for music-making. A photographic overview of the devices used by the participants in this study can be found in Figure 3. Table 1 lists the hardware and software tools, provides a brief description of each setup, and presents the participants' self-reported musical styles, which ranged from experimental electro-acoustic improvisation to Afro house.

**Figure 3:**
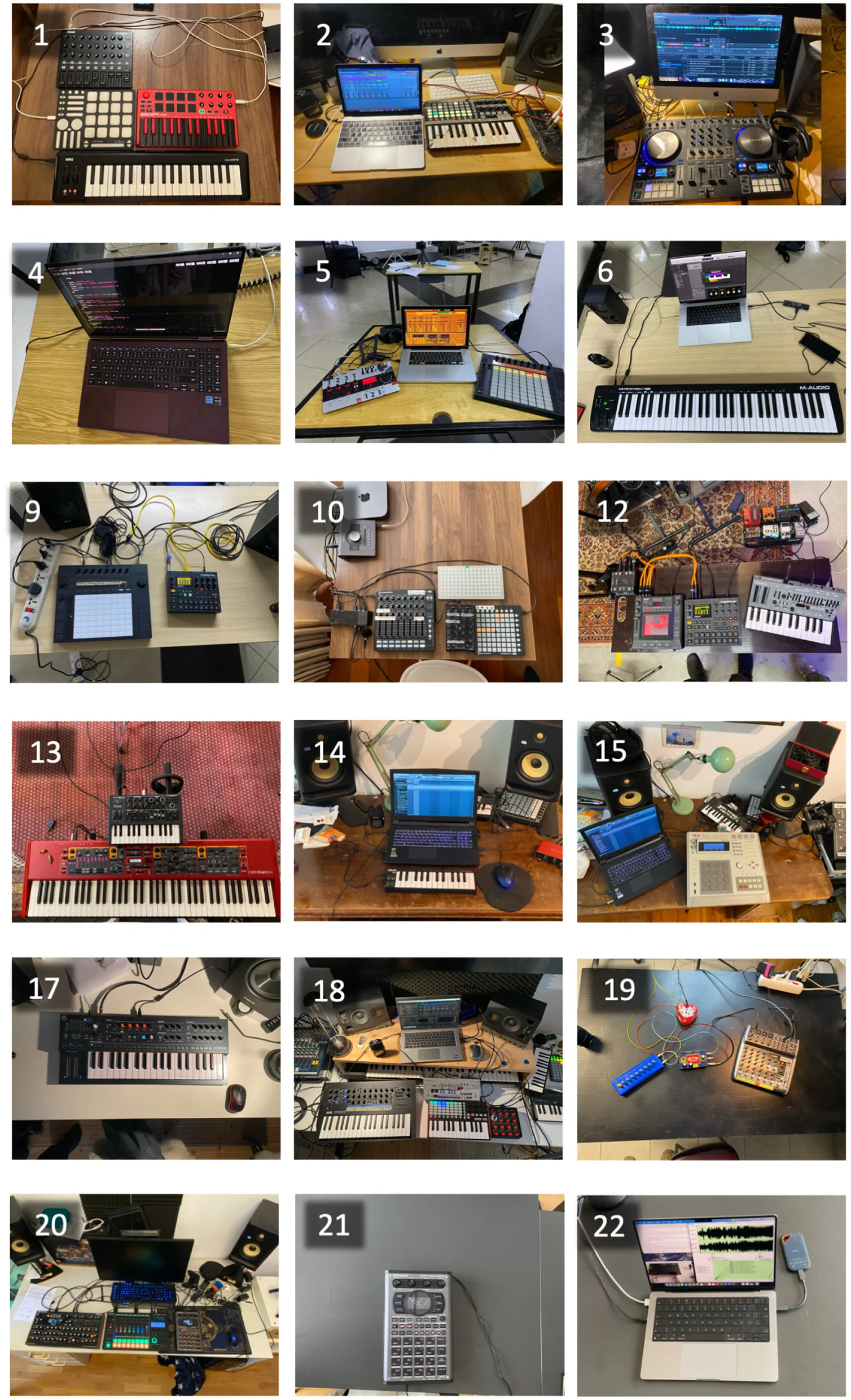
An overview of all devices used by the participants.

From a componential viewpoint, prevalent interface element types are keyboard keys (both for instrumental and computer keyboards), knobs (both encoders and potentiometers), pads (drum pads and clip launchers), faders, small and large displays, and buttons. Several devices contained knobs organized in grids, and pads were also frequently combined into grids. Less frequently used interface elements were jogwheels, touchscreens, and camera interfaces. From a software perspective, four participants used Ableton Live both with and without the graphical user interface. Other digital audio workstations, as well as DJ software, were also used. Two participants used the programming languages Sonic Pi and Pure Data. Several participants used hardware sequencing. The electronic music performers in our dataset used pre-produced tracks (stems) but also sequencers, MIDI or audio loops and audio samples during their performances. These processes share a common trait: once the process is started or modified, the effect may unfold at a later moment in time, giving the performer no immediate auditory feedback, whether the activation or modification was successful. Our participants have exhibited different behaviors that tackle this challenge, mostly using visual feedback in lieu of vibrotactile or sensorimotor feedback. Participant 18 resorted to using visual feedback from the volume meter on the laptop display to resolve what they called the “classic pad problem,” where a sound unfolding in time cannot be recognized initially. Similarly, participant 19 addressed a problem with a distorting step on their DIY step sequencer by following the step activation light with their eyes to identify the step to be altered. Furthermore, we found indicators that performers map important parameters such as gain or volume to the outside of a device or a grid layout. An example of this can be seen in the mapping visualization of participant 1 in Figure 1C.

### 3.2 Scan path analysis

An overview of scan paths during the performances is given in Figure 2. The areas of the scan path that consist of several fixation circles highlight areas on the interfaces that are frequently visually attended. Recurring gaze patterns, and by extension patterns of visual attention, can also be visible on the scan paths. While it is possible to display the absolute sequence of fixations on areas of interest, the scan paths in Figure 2 inform about general tendencies: the scan paths of some performances exhibit certain geometric shapes that emerge as the general gaze tendencies during the performances.

Across setups, there are devices where the majority of fixations are concentrated on a distinct part of the interface. On larger visual displays, such as laptop screens, this is particularly interesting when participants share the same software: the DJ fixates on the waveform display (Participant 3), both producers fixate on FL Studio's sample browser (Participants 14, 15), and users of Ableton Live fixate on the channel strips in the session view and the device view.

Gaze patterns from setups with more than two devices (Participants 1, 5, 10, 12, 18, 19, 20) seem to fall into one of two categories. For the first category, the devices are arranged in a line, making it hard to pass qualitative judgment on the scan paths between devices (Participants 12, 19, 20). For the second category, scan paths seem to originate from and return to a nexus area across one of the devices (Participants 1, 5, 10). Participant 18's scan path, however, presents more interconnected fixation areas than from Participants 1, 5 and 10. Interestingly, in those cases, devices with grids of knobs often appeared as nexus areas.

For the gazing pattern of participant 5, a triangular shape emerges. However, the bottom side of this triangle, highlighting the connection between Kore 1 and Push 1 controllers, is less pronounced than the sides going from the laptop screen to either of them. This shows that visual attention rarely shifts from one controller to another without passing by the screen. For the DJ performance (Figure 2, 3), a triangular gazing pattern appears as well: the triangle highlights the two most looked-at locations of the setup: the mixer section on the DJ controller, as well as the waveform display and master control panel on the computer screen. The scan path seems to create a triangular pattern because of the waveform display: as the performer looks up from the mixer section, the gaze follows the waveform moving from left to right, until at one point the participant looks at the mixer section again.

The scan paths reveal that for all performances, participants fixated on their live-electronics setups. The scan path analysis shows which elements of a live-electronics setup receive visual attention. However, this does not paint the full picture. In order to obtain more detailed insight, we have to examine fixation metrics and the interview transcripts.

### 3.3 Fixation metrics

Table 1 shows the Fixation Count (*n*_*F*_), the Total Fixation Duration (*t*_*FD*_), the Average Fixation duration (μ_*FD*_), and the Ratio of Performance Duration (ρ_*PD*_) per device. A Mann–Whitney *U*-test on all devices with *n*_*F*_>0 indicated that the Average Fixation duration μ_*FD*_ was significantly greater for laptop and monitor devices compared to the remaining devices (*N*_*Laptop*/*Monitor*_ = 9, *N*_*Other*_ = 35, *U*_*Laptop*/*Monitor*_ = 254.0, *z* = 2.808, *p* = .002, *r* = .423). This shows that performer fixations on laptops and monitors tend to be longer than on other devices.

Devices that contained one or more grids of knobs had the highest fixation count *n*_*F*_ in eight of the performances (Participants 1, 2, 3, 10, 12, 13, 18, 20). However, a second Mann–Whitney *U*-test on devices that contain grids of knobs compared to those without did not exhibit a statistically significant difference regarding μ_*FD*_.

Furthermore, the fixation metrics complement the scan path analysis. On average, 94.6 % of fixations occurred on the surface of devices of the LES (SD = 0.043). For participants 12 and 20, the device in the middle of the LES (the Elektron Digitakt; the Roland MC-707) has the highest fixation count *n*_*F*_ of their respective LES. Participant 19, however, fixated most often on the DIY sequencer on the left. The devices of participants 1, 5, and 10, which were previously described as nexus areas, also have the highest fixation count *n*_*F*_ within their LES.

### 3.4 Thematic analysis

The identified themes of eye gaze in live-electronic performance can be grouped in two main categories: the looking–listening interplay and visual information. Looking–listening interplay encompasses the themes of *looking because an element represents a sound* and *looking to substitute or aid hearing*. Visual information encompasses *looking for visual feedback of a system* and *looking for targets in hand-eye coordination*. Figure 4 shows the number of performers that delivered statements that address the given themes alongside minor themes. While the aforementioned themes explain *why* someone looks at their devices during a performance, the remaining minor themes address why someone might look away from them. Note, due to the laboratory conditions during recording, participants did not have the opportunity to look at an audience.

**Figure 4:**
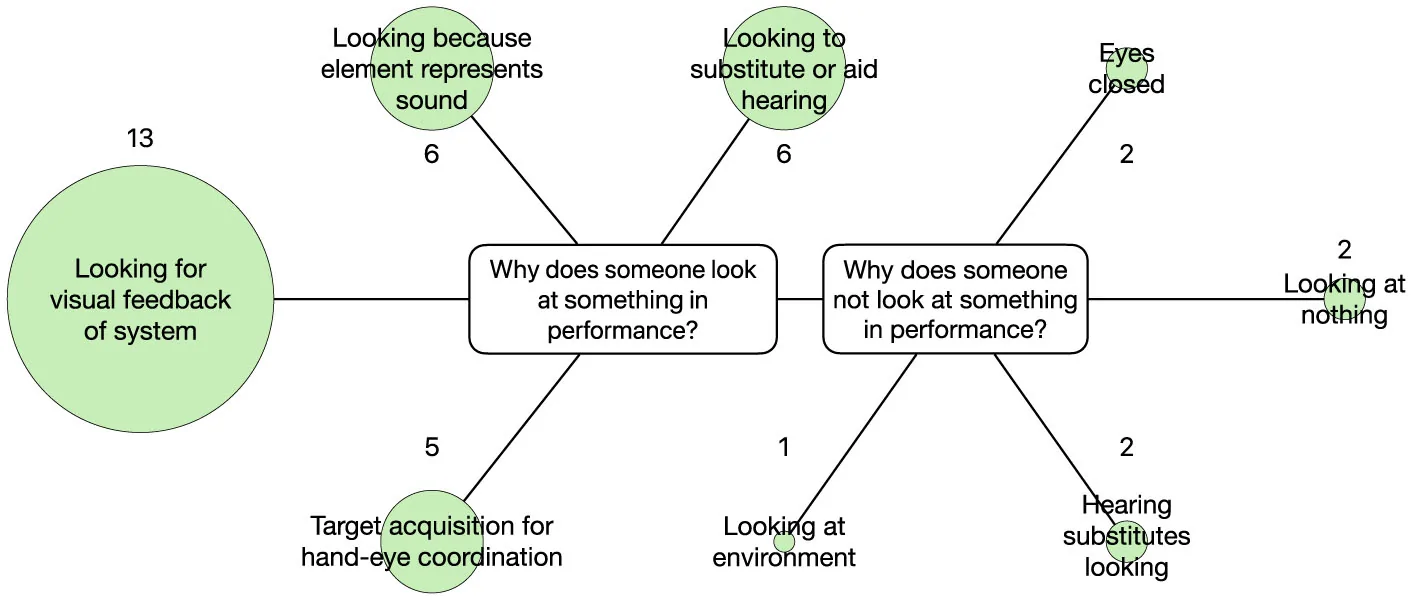
Themes identified pertaining to why a performer may or may not look at an element during performance. Radius of green circles and number reflect number of participants that addressed these themes during the interview.

#### 3.4.1 Looking because element represents sound

A frequently reported theme (*n* = 6) was looking at an element because the element represents a sound, sound source, or sound process. While this is sometimes hard to separate from target acquisition for hand-eye coordination, these are distinct statements that suggest that performers sometimes look at a certain element when thinking about the associated sound. Participant 1 describes such an event: A set of eight pads on their AKAI MPK Mini loads different drum samples into an arpeggiator when pressed. Once a sample is loaded into this arpeggiator, the need to look at it for target acquisition subsides. Interestingly, participant 1 still looked at the pad fairly frequently while operating on the rotary potentiometers that control the arpeggiator. They commented on this particular behavior: “Ich schaue immer wieder auf das Pad, obwohl ich das nicht dracke, aber da kommt der Sound halt raus (Translation: I am always looking at the pad, even though I won't press it, but that's where the sound originates from).” They also refer to the pad as a “Klangquelle” (sound source).

For participant 10, looking at their hands-on interface elements can be a way of bringing them to the foreground of the mental representation of the soundscape. It reminds them of what they are doing with the right hand; sounds that are related to the actions of the hands will be more “clear” and “prominent.” While they emphasize that they do not actually need to look in order to hear, they describe that it helps them “focus on specific things.” Especially with processes like sequencers and loopers, participant 10 mentions how the visual scanning of their setup helps them decide what to “add here, or take away here, or change” by reminding them of all the musical elements that are currently engaged. In a similar line of explanation, participant 18 mentioned how, in a moment of planning what to do next, they used their eyes to glance around their interfaces. They orient themselves by asking, “What am I doing, playing? What am I turning there?” (German: “Was mache ich, spiel ich? Was drehe ich da?”). Participant 20 reiterated what participant 10 described as looking to bring musical elements into focus despite not having to look. They mentioned that they sometimes only listen to the sounds, they can even close their eyes, but sometimes there are moments where they “stare at the sound” (German: “den Sound anstarren”) trying to hear it.

#### 3.4.2 Looking to substitute or aid hearing

Some software and hardware tools for electronic music making may come with some redundancy between visual and auditory information. Participants 3 and 22 mentioned checking the waveform for information about the volume of a sound file, and participant 5 for tonal content, which substitutes the act of hearing with looking. It has also been reported how looking at what the hands are doing reportedly aids participants' hearing by bringing certain sound objects into focus, as in the case of participant 10, perhaps through mental representations of the interface elements. Participant 18 describes an interesting moment when they moved a knob trying to increase a lowpass filter cutoff frequency. When this did not yield any audible results, they resorted to looking at the screen in order to figure out why the filter is not active. Here they realized that the filter indeed is active and that the frequency had just not been increased to such a degree that the change became noticeable. Similarly, they reported an instance where they had triggered a pad sound but, due to the long attack of the pad, had to check the volume display on screen to validate whether the pad had actually been activated. They called this the “classic pad problem” (German: “Das klassische Padproblem”). It seems plausible that such an issue might occur frequently for performers that work with slowly unfolding samples.

#### 3.4.3 Looking for visual feedback of system

The most often addressed theme was looking at an element for gaining information about the current state of the system. This is important, because live-electronic music performance is complex, and contains potential for mishaps. When discussing a moment something went awry during the performance of participant 2, they emphasized the role of the laptop in getting back into the proper mindset or point within the performance: “So, when I'm not in the zone, the laptop is kind of like, ‘[...] this is where you are.' ” Participant 3, the DJ of the dataset, mentioned using track information, the beats-per-minute display, graphical user interface elements for live synchronization of tracks, and the waveform. They check the waveform for synchronization, volume, and content information. In a similar line of reasoning, participant 5, who operates a live performance process of loading audio samples into a granular buffer, uses the visual feedback of a waveform display as a means of orientation: “[...] sometimes I can tell by the waveform what's in there. I'll see there's a base note in there. [...] So I'll have to see the waveforms to take decisions. [...] Okay, there's a long tail, I can slowly sweep into the tail, empty the buffer, and add a new layer.” Part of this sequence of performance actions can be seen on the left side of Figure 5. Here, the top part shows a repeated pattern of changing a synthesis parameter and examining or verifying the system's changes through the graphical user interface. Participant 22 also used a waveform display to estimate both the correct position for granular playback and the correct loudness of the position. Visual displays can also play a role in providing semantic, textual information during a performance. For participant 4, who performed live coding, their estimate of the most looked-at part of their setup was the left area of the computer display, as “that's where the code is.” In this study, laptops and monitor devices exhibited a wide range of Average Fixation Durations (μ_*FD*_ from 0.351 to 0.678 seconds, see Table 1). At the same time, they were used in a multitude of contexts, in conjunction with different numbers of other devices and presented varying qualities of information. These may all affect the duration of fixations on a device.

**Figure 5:**
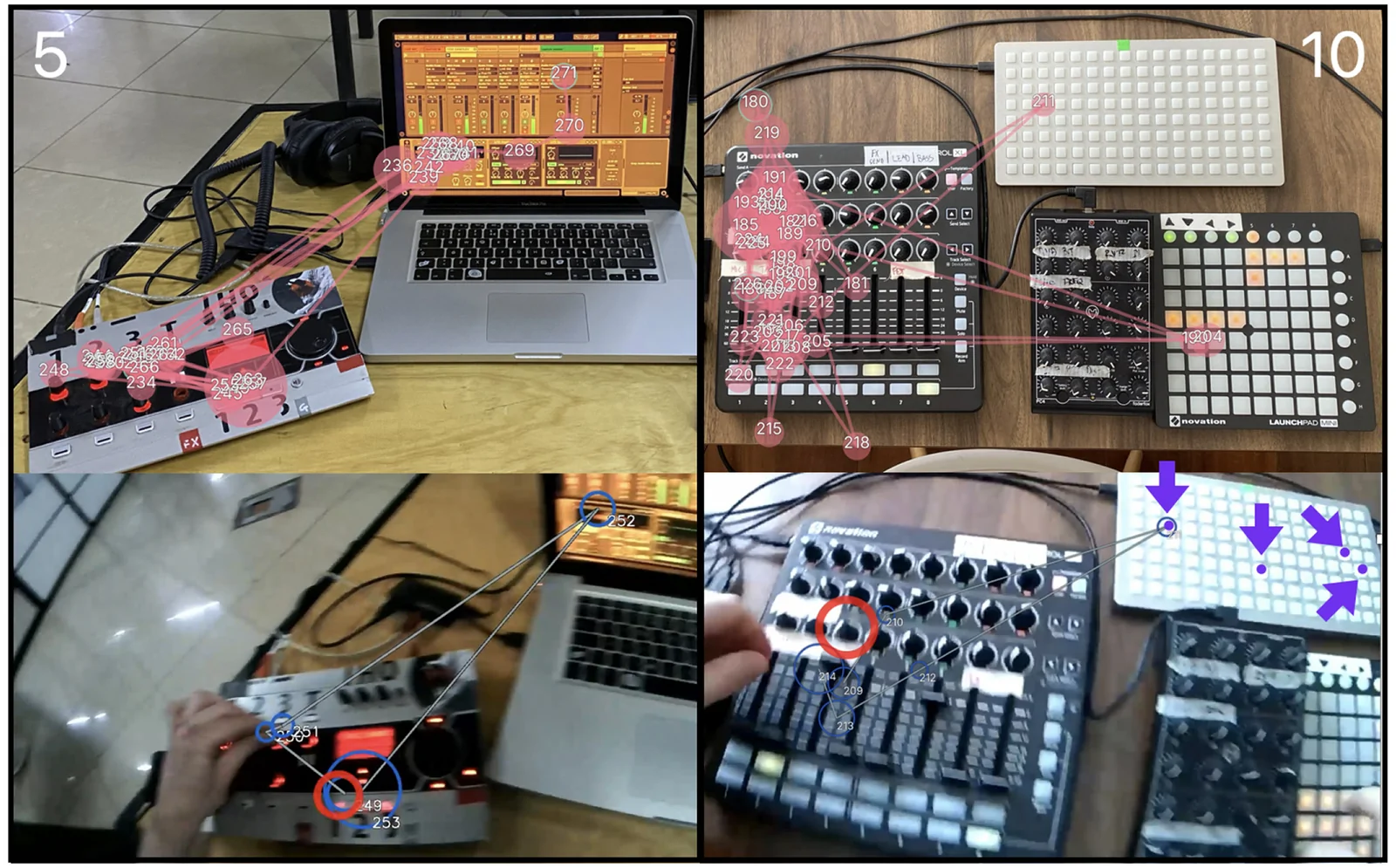
Moments of participants 5 (left) and 10 (right) looking for visual feedback of a system using different forms of visual display. Top half presents an excerpt of each moment with fixations (pink circles) overlaid on top of a reference image (left: 22.1 s, right: 23.2 s). Bottom half of the image shows a shorter excerpt of the same moment (left: 6.3 s, right: 5.1 s) from the FOV camera with fixations (blue circles) and momentary point of gaze (red circle) mapped to it. Left: with one hand on the KORE 1 controller, Participant 5's gaze continuously moves back and forth between the controller and the graphical user interface, which shows granular synthesis parameters such as the playback position relative to the sample. Right: while operating on and gazing at the Launchcontrol XL (left hand), Participant 10 briefly gazes at one of the active, glowing cells of the monome grid controller (purple arrows).

But not only visual displays serve some kind of visual feedback. A performer may also derive the state of a system parameter from the relative position of an interface element. Participant 18 describes a case where they looked at a control element in order to gather information on the state of the parameter they are controlling. In this case, it was an encoder that had a local display of the virtual controller parameter state in direct proximity around it. Participant 20 also looked at an interface element to estimate the position of a filter cutoff. In addition, they explained that light indicators help them verify which performance elements are active just from a glance. In a similar line, participant 17 described an event during the performance where they changed a parameter using an interface element and looked at the visual display to verify changes to the parameter state. Participant 10, during a section of the performance mainly engaged in performing on the Launchcontrol XL and the Launchpad Mini controllers, suddenly moves their gaze from the Launchcontrol XL to one of the active, glowing cells on the monome grid controller, then immediately gazes back at the Lauchcontrol XL (right half of Figure 5). As participant 10 puts it, “There's so many layers here. I'm not even sure all the time where everything is coming from.”

A comparison between the Average Fixation Duration μ_*FD*_ of the laptop of participant 5 and the monome grid of participant 10 (see Figure 5) reveals a potential link between the amount or quality of information presented on the interface and the time it takes to extract it. While the laptop with its graphical user interface and dense, symbolic information had a μ_*FD*_ of 0.683 s, the simple visual interface of glowing buttons of the monome grid had a μ_*FD*_ of only 0.363 seconds (see Table 1).

#### 3.4.4 Looking for targets in hand-eye coordination

Five participants provided statements on the use of their gaze to orient their hands toward a target to perform an action. Participant 1, for example, described such an action. During the performance, they found themselves in a position where the individual gain parameters for their software instruments were already at their maximum. Wanting to increase the loudness nonetheless, they looked at the master gain in order to evaluate how much headroom they could receive for the individual gain parameters from increasing the master gain while simultaneously decreasing the individual gain parameters. Upon evaluating this, they performed the trade-off. Participant 13 also touched on this subject when discussing switching from one keyboard instrument to the other during a transition from one part in their performance to another. They explained that in order to prepare for the verse, they look at the position where they are going to play the chords soon after. After arriving at the starting position, they had the remaining chord positions firmly under their control without having to look further (the German phrase they used, “dann habe ich die danach auch im Griff,” can refer to both having a situation under control and to having something within the act of grasping). In contrast to this, participant 14 explained that when playing chords, contrary to a “real pianist,” who is able to make out the position in relation to the keyboard by touch or proprioception, they look at the lowest note of the chord they are going to play first and build the chord from there. Participant 1, a classically trained pianist, fixated on the keyboard interfaces only a few times, as can be seen in Figures 1, 2. One example where they do fixate on a key of the keyboard mapped to their bass instrument rack is pictured in Figure 1B.

#### 3.4.5 Speed of eye movements

In addition to the previous themes of *why* participants looked at their devices during performance, three participants also commented unprompted that they perceived their eye movements to be fast. Participant 18 commented that they were not aware of the speed with which they looked around (“Krass, wie schnell hin- und her geguckt wird. Das war mir gar nicht so bewusst”). Participant 4 commented on the speed of their gaze while mimicking the movement with their fingers (“It is moving so fast”). Participant 22 commented that their gazing is moving fast from place to place (“Meine Blicke, ziemlich schnell von Ort zu Ort”). This demonstrates that participants are unaware of certain automated aspects of body movements during their performance.

## 4 Discussion

The present study provides a bird's-eye view on the role of eye gaze in the field of live-electronic music performance. From performer statements in a stimulated recall interview, we have compiled and documented two main categories of performer-reported functions of gaze during a live-electronic music performance: visual information and looking–listening interplay. Within these categories, gaze can be used to 1) receive visual feedback about the state of an LES, 2) locate a target and guide the hand toward it, 3) aid memory retrieval of a process associated with the visual target, and 4) aid listening to a process associated with the visual target. Another obvious function of eye gaze in any music performance is to extract information from a score. We have excluded one performance from the analysis due to the performer playing with a score. While this is a common and documented practice in academic electronic music (Emmerson, 2006; Grund et al., 2024; Hofmann and Stojak, 2026), the fact that we had only one such performance discouraged us from including it in this study. We recommend that future research investigates the effect a score has on a performer's gaze in live-electronic music in a separate study.

Visual attention can be the result of task-oriented, top-down processes or of stimulus-driven, bottom-up processes (Kaya and Elhilali, 2017; Hayhoe and Ballard, 2005). The results of the scan path analysis revealed that across all performances, a great deal of visual attention was allocated to the live-electronics setups. Hayhoe and Ballard (2005) argue that, when fixations pre-dominantly fall on task-relevant objects, the observer necessarily must know which objects are task-relevant. We have seen that most of the fixations during a performance fall on areas of interest that are task-relevant: the devices of the LES. Furthermore, clusters of fixations and saccades between devices form geometric shapes within the scan paths that may reflect dependencies between devices or performance patterns. This suggests that gaze during a live-electronic performance is at least in part governed in a top-down manner, driven by the performer's intention. Connecting the geometric shapes of the scan paths with the performers' intention was possible because the performers revealed their workflow to us during post-performance stimulated recall interviews.

Visual displays were consistently looked at. As visual displays in our study provided momentary visual feedback of the current state of the system as well as an overview or representations of sound, software and hardware objects, and relevant sound or performance material, it is unsurprising that performers choose to perform with them. Their capabilities touch on three out of four major themes identified in the interview transcripts: visual feedback, sound representative elements, and looking as a mechanism to aid hearing. Furthermore, visual displays were identified as an aid in performance scenarios that required management of different time scales. While visual displays are of limited use when a performer uses their gaze for hand-eye coordination to a target element, they have proven useful for mentally connecting performance actions on a target element—such as turning a knob—with their effects on the system side. While this is useful for interface elements with a static mapping, it becomes even more relevant for those with dynamic mappings, such as the effect-dependent control knobs on the Roland SP404 or the rotary encoders on the Arturia Minifreak or DJ Techtools MIDI Fighter Twister. Visual displays also allow a performer to engage with a setup on a semantic level, either by receiving information via parameter names and values or by providing textual information, for example, during live coding. Semantic processing may be one of the driving factors of the moderately greater μ_*FD*_ on visual display devices compared to other devices. Our interpretation, based on participants' statements, is that these visual displays often consist of dense and abstract information in a symbolic form, such as text, or expressed through object properties, such as the relative spatial position of a playhead to a waveform or a red-clipping gain meter. Extracting this information, while simultaneously relating it to the auditory information, can be a challenging task that warrants longer fixations, as longer fixations can be an indicator of increased mental load according to Goldberg and Kotval (1999).

Devices that contain grids of knobs were not only prevalent in our study as a whole but were often the most looked at device of their respective LES, from the mixer section on the DJ setup to MIDI controllers with partial or full coverage of grids of knobs. Interestingly, it was observed that having a grid of knobs present on an interface sometimes coincides with it appearing as a nexus in the scan path analysis. This is of course, not disconnected from the musical context these devices are used in, nor from the mapping, but it still warrants a deeper analysis, as it is unclear whether grids of knobs are looked at much (a) because they contain the most important performance parameters, (b) because they contain a lot of parameters on limited space, or (c) because the lack of salient visual features forces a performer to search for relevant knobs for a prolonged duration compared to other interface elements. We hypothesize that a combination of these assumptions is true, as we have examples of participants mapping important performance parameters, such as the master gain, to the border of the grid. Presumably, this is for ease of access; however, one should also consider the influence of performance culture on mapping behaviors, such as through the mixing-board paradigm. Future research should therefore focus on investigating mapping behaviors, mapping strategies, and their effect on the learnability, speed, and accuracy of a performance with different musical mappings.

Gathering visual information is an important function of gaze in live-electronic music. This is corroborated by the facts that thirteen participants touched on the subject of visual feedback in the interviews. Every single LES we covered had at least one device with a display or an LED that presented some information about the state of the musical system. Similarly, even control interface elements such as knobs have visual markers that are used by performers to infer the state of the system. In several cases in our study visual displays, however, managed to provide more meaningful, sound-related information than visual markers on the control interface element and sometimes necessary confirmation of activity or extent of sound manipulation. Magnusson describes a difference in embodiment and incorporated knowledge between acoustic and screen-based instruments (Magnusson, 2006): while in acoustic instruments it is motor memory that is a deciding factor in learning, for screen-based instruments it is what Magnusson calls “visual and procedural memory.” These considerations are not limited to what Magnusson refers to as screen-based instruments but can be extended to most of the devices in this work, with the notable exceptions of keyboard and drum pad interfaces. Visual feedback untangles the complexities of live-electronic music performance. It helps participants plan ahead, manage sound parameters that have no immediate effect on the sound, verify the activity of elements, and provides a safety net for performers in case of performance mishaps.

Another frequently described theme for visual information was to use gaze to acquire a target for interaction and guide the hand toward it using hand-eye coordination. It is surprising that not more participants commented on this widely documented phenomenon (Ballard et al., 1992; Coudiere and Danion, 2024). Perhaps hand-eye coordination is a process so natural, so automated to most performers, that they could not mention it at all. When performers did mention it, it appeared mostly in context with some form of transition. Here, it would be interesting for future research to systematically test different interface designs for possible effects on the fixation-to-touch duration. Regarding automaticity, however, gaze tracking did reveal some automated processes, as participants commented on the speed with which their eyes move between areas of interest. Due to the different technological means and stylistic expressions present, it is not possible to draw conclusions about differences in gaze metrics between novices and experts, as they are frequently reported in performance research (Hayhoe and Ballard, 2005; Mitchell et al., 2020). Future research should investigate this question with a fixed setup.

When a performer is not able to identify an audible effect that changing a control parameter has, this constitutes an interesting case where gaze may substitute hearing. Most notably, performers stated that looking at an interface element helps bring it into focus during a performance. In a live-electronic music performance, there are often multiple processes of sound production and manipulation active in concurrence. As attention is a limited resource, it is important to manage it effectively. Looking at interface elements to selectively bring them into focus may be a mechanism suitable for attention management. We theorize that in memory, conceptual, visual, and spatial representations of an LES are integrated with auditory expectations. Visuospatial attention, such as looking at interface elements, may help with the retrieval of these internal representations. Findings from other studies suggest that spatial selective attention modulates the processing of visual stimuli (Mangun et al., 2001) and that scene representation in visual memory supports remembering visual and spatial information about (concrete) objects within a scene (Hollingworth, 2005; Ferreira et al., 2008; Johansson and Johansson, 2014). This supports our assumption that in the case of looking at interface elements visuospatial attention supports auditory information retrieval and processing during live-electronic music performance.

### 4.1 Limitations

For the design of this study, we employed a wide definition of live-electronic music performance to purposefully include diverse practices. However, this diversity of practices also led to a diversity of themes that performers reported. Future studies might benefit from a more focused selection of participants. A more focused selection could also improve the gaze analysis, as it could make the results more comparable. Furthermore, we identified themes of eye gaze in live-electronic music performance based on performer statements. While these results are situated in an ecologically grounded setting, they are based on qualitative, retrospective self-reports, and therefore hard to generalize and prone to bias. We cannot verify whether gazing at a visual target actually helps with retrieving the internal representation of a system or with the act of hearing. We therefore recommend future research to systematically investigate aspects of eye gaze in live-electronic music performance while acknowledging the musical nature of these systems. Potential studies could quantitatively investigate the role of eye gaze and its relationship to auditory attention, mental representation, and musical decision-making.

## 5 Conclusion

This study provides qualitative insight into the roles of eye gaze in live-electronic music performance. From stimulated recall interviews using gaze tracking recordings of live-electronic music performances as the stimulus, performer statements were extracted and analyzed to identify common themes. The findings suggest that eye gaze in a live-electronic music performance is not only used to guide hands toward an interface of a musical system or to receive information about the system's state. Performer statements imply that the act of looking directs auditory attention to a part of the musical system associated with the visual target and might even help retrieve mental representations of the system. This has implications for the design of new musical devices, as visually cluttered or unintuitive interface designs might impact this process.

## Data Availability

The datasets presented in this study can be found in online repositories. The names of the repository/repositories and accession number(s) can be found in the article/supplementary material.
